# Aging-related markers in rat urine revealed by dynamic metabolic profiling using machine learning

**DOI:** 10.18632/aging.203046

**Published:** 2021-05-19

**Authors:** Dan Shi, Qilong Tan, Jingqi Ruan, Zhen Tian, Xinyue Wang, Jinxiao Liu, Xin Liu, Zhipeng Liu, Yuntao Zhang, Changhao Sun, Yucun Niu

**Affiliations:** 1 National Key Discipline Laboratory, Department of Nutrition and Food Hygiene, School of Public Health, Harbin Medical University, Harbin, PR China; 2 Department of Nutrition and Food Hygiene, School of Public Health and Management, Chongqing Medical University, Chongqing 400016, PR China; 3 Department of Epidemiology and Biostatistics, School of Public Health, Harbin Medical University, Harbin, PR China

**Keywords:** aging, metabolic trajectories, biomarkers, time-series, machine learning

## Abstract

The process of aging and metabolism is intimately intertwined; thus, developing biomarkers related to metabolism is critical for delaying aging. However, few studies have identified reliable markers that reflect aging trajectories based on machine learning. We generated metabolomic profiles from rat urine using ultra-performance liquid chromatography/mass spectrometry. This was dynamically collected at four stages of the rat’s age (20, 50, 75, and 100 weeks) for both the training and test groups. Partial least squares-discriminant analysis score plots revealed a perfect separation trajectory in one direction with increasing age in the training and test groups. We further screened 25 aging-related biomarkers through the combination of four algorithms (VIP, time-series, LASSO, and SVM-RFE) in the training group. They were validated in the test group with an area under the curve of 1. Finally, six metabolites, known or novel aging-related markers, were identified, including epinephrine, glutarylcarnitine, L-kynurenine, taurine, 3-hydroxydodecanedioic acid, and N-acetylcitrulline. We also found that, except for N-acetylcitrulline (*p* < 0.05), the identified aging-related metabolites did not differ between tumor-free and tumor-bearing rats at 100 weeks (*p* > 0.05). Our findings reveal the metabolic trajectories of aging and provide novel biomarkers as potential therapeutic antiaging targets.

## INTRODUCTION

Aging is an inevitable physiological process that is accompanied by a broad range of functional impairments and metabolic disorders [1]. Several reports have provided evidence that many factors could be drivers of aging and age-related disorders in various species, including *Drosophila melanogaster*, *Mus musculus*, and humans [2–6]. Despite considerable efforts and the development of rich aging theories, the underlying mechanism of aging is still largely unknown, in part because of the complexity of the aging process with remarkable variation and changes in systemic metabolism.

Untargeted metabolomics technology was used to characterize a large panel of small molecules in the biological system involved in the aging process. This could reflect the by-products of metabolism or other damage forms that accumulate in old organisms and provide clues to reveal the development of the aging process. Thereby, aiding to identify reliable biomarkers for predicting biological age [7–9]. Analysis of metabolic profiles in young and old mice revealed aging-associated changes, including nutrient sensing and lipid and amino acid metabolism [10, 11]. In addition, metabolomics-based and lipidomics-based investigations of centenarians revealed phospho-/sphingolipids as putative longevity markers [12]. Another analytical study of the plasma metabolome of 2,327 aging individuals using liquid chromatography/mass spectrometry (LC/MS) identified a different set of metabolites related to longevity, including isocitrate, bile acid, and taurocholate [5]. Although much work has been done to identify the aging biomarkers in a broader range of species, only a few studies have been conducted in clinical practice. The reason may be that most biomarkers fail to dynamically reflect the effectiveness at different life stages. Furthermore, there are inaccuracies in screening robust aging markers for both sensitivity and specificity, especially for observations that mostly focus on cross-sectional and short-term associations of metabolites with age. Therefore, additional longitudinal studies using metabolomics technology are needed to dynamically identify and screen aging-related markers based on cutting-edge statistical methods, such as machine learning.

In this study, we dynamically performed a high-throughput metabolomics-based investigation in two rat cohorts, starting from the early adult phase to the old age. We sought to identify circulating urine metabolites associated with aging in combination with four algorithms: VIP, time-series, LASSO, and SVM-RFE. We discovered unique metabolic compounds related to aging, including glutarylcarnitine and 3-hydroxydodecanedioic acid, which may be good predictors of aging when considering both sensitivity and specificity. Furthermore, we found that, except for N-acetylcitrulline, none of the identified aging-related metabolites were related to tumor occurrence in rats at 100 weeks.

## RESULTS

### Food intake, body weight, and urine metabolic profiles with aging

**Figure 1:**
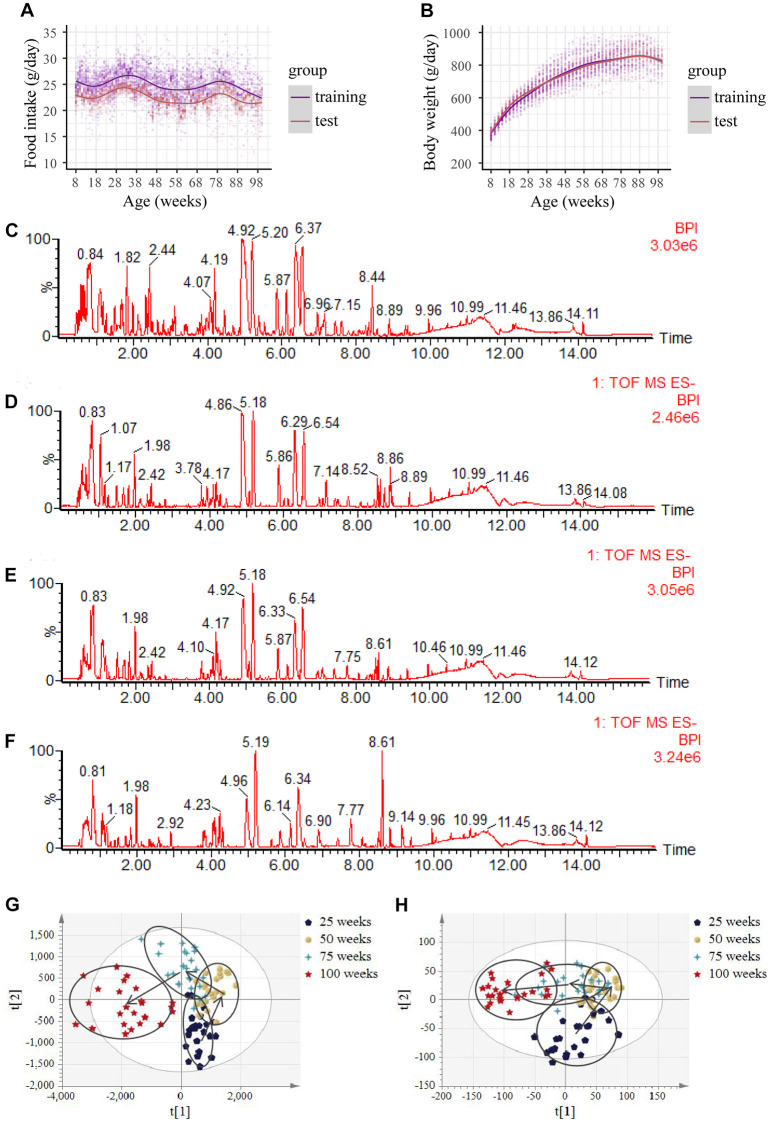
**Food intake, body weight, base peak intensity chromatograms and urine metabolic profiles with aging.** (**A**) Food intake in both the training and test group. (Initial sample size: *n* = 60 in the training group; *n* = 30 in the test group). (**B**) Body weight in both the training and test group. (Initial sample size: *n* = 60 in the training group; n=30 in the test group). (**C**–**F**) Representative negative base peak intensity (BPI) chromatograms at the age of (**C**) 25 weeks, (**D**) 50 weeks, (**E**) 75 weeks and (**F**) 100 weeks of rats using UPLC-Q-TOF-MS in electrospray ionization negative (ESI−) ion mode in the training group. (**G**–**H**) PLS-DA scores plots of urine obtained from the UPLC-Q-TOF-MS with age in (**G**) left: the negative mode, and (**H**) right: the positive mode in the training group. (*n* = 24 each time point).

In this study, the training group included 60 male Wistar rats, and the test group consisted of 30 male Wistar rats. They were fed pelleted chow starting from 8 weeks. The food intake and body weight trajectory of rats with aging in the training and test groups are shown in Figure 1A and 1B. Food intake in both the training and test groups fluctuated with increasing age (Figure 1A). The body weight in the training group increased in the first 58 weeks with the increase in age. Then slowly stabilized, and started dropping from 93 weeks (Figure 1B). The downward trend continued with the increase in age (data are not shown), suggesting that the rats entered the fragile state. Similar results were observed for the test group (Figure 1B). Moreover, at 100 weeks, a total of 43 rats died naturally (data not shown), further indicating that the rats entered the aging stage at 100 weeks. Compared with the test group, the food intake, on average, in the training group was lower (*p* < 0.05), but body weights displayed no statistical difference (*p* > 0.05) (Figure 1A and 1B).

During the study period, the urine of rats in the training group was dynamically collected for metabolomic analysis, and detected by ultra-high-performance liquid chromatography-tandem quadrupole time-of-flight mass spectrometry (UPLC-Q-TOF-MS). Principal component analysis, including a quality control (QC) sample and an experimental sample of time data of the training group in the positive or negative modes, was performed. The QC results were tightly clustered (Supplementary Figure 1A, 1B), suggesting the stability of the system and the reliability of the data. Representative chromatograms of the base peak intensity at four time points are displayed in Figure 1C–1F. Using the “80% rule” filter criteria of primary data in the training group, we derived a list of 10,729 metabolites in the negative mode and a list of 27,962 metabolites in the positive mode for subsequent multivariate statistical analysis. The partial least squares-discriminant analysis (PLS-DA) score plots from both electrospray ionization (ESI)+ and ESI− based on all raw metabolites revealed a perfect separation trajectory in one direction, at different time points, and with increasing age (Figure 1G and 1H). In total, the results suggest that metabolic remodeling occurs during aging, leading to alterations in urinary metabolites.

### Metabolite aging trajectories based on a time-series analysis

**Figure 2:**
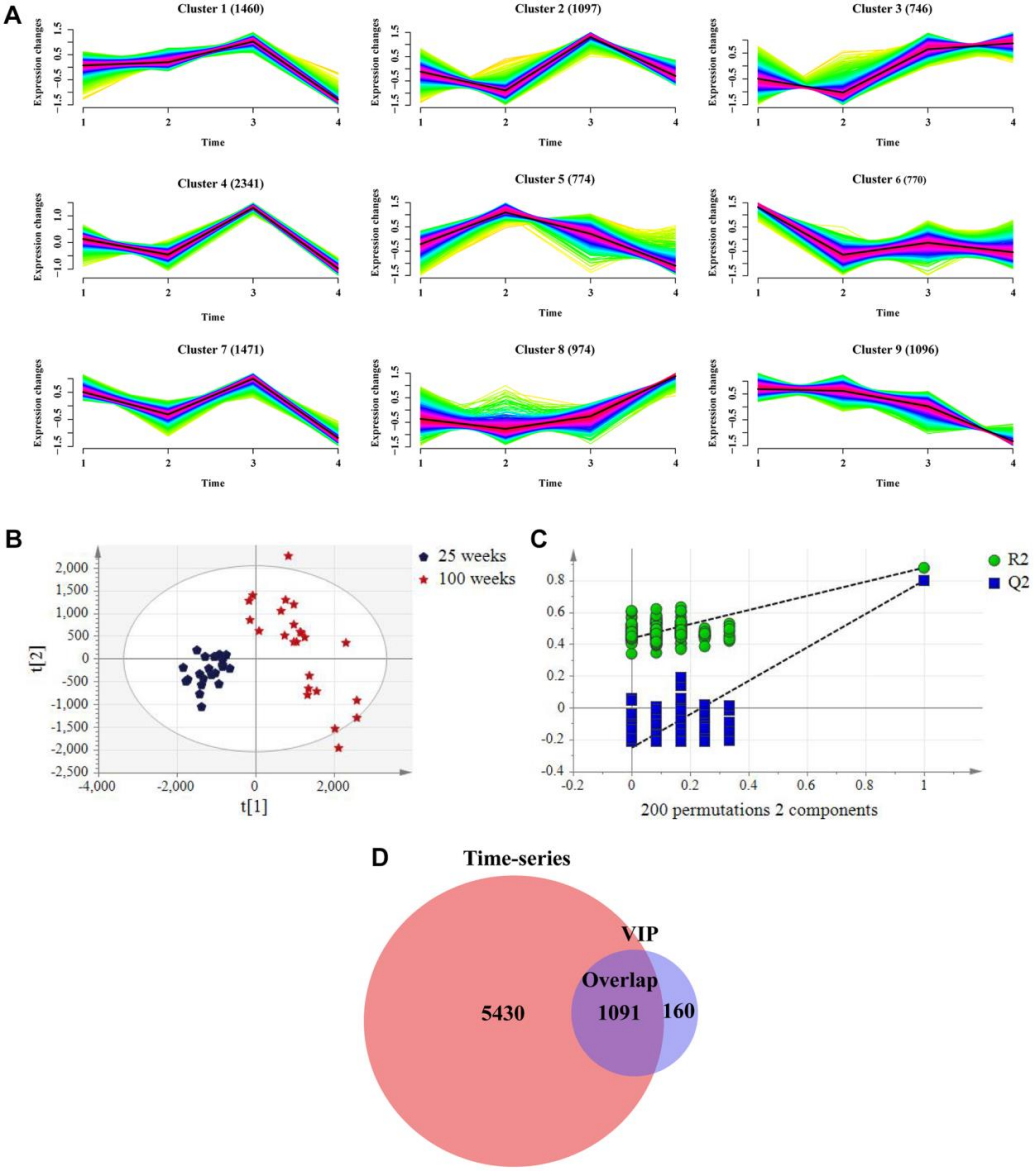
**Screening metabolites in combination with time-series analysis and variable importance in the project (VIP) value in the negative mode in the training group.** (**A**) Metabolic aging trajectories. Fuzzy c-mean clustering of all 10729 metabolite abundances. Average trend of clusters is shown as a black line. (**B**) PLS-DA scores plots of rat urine at 25 weeks and 100 weeks. (ESI−, R2Y= 0.881, Q2 = 0.80). (*n* = 24 each time point). (**C**) Permutation test with 2 components of PLS-DA score plot. (**D**) Venn diagrams demonstrated the overlap of metabolites in combination with two algorithms.

To dynamically identify age-related metabolic trajectories, we performed a time-series analysis and predefined nine clusters based on metabolite aging trajectories in the negative and positive modes (Figure 2A and Supplementary Figure 2A). Considering as many age-related metabolites as possible, we selected clusters 1, 3, 5, 7, 8, and 9 containing 60.8% of the metabolites in the negative mode and clusters 3, 4, 7, and 8 contained 71% of the metabolites in the positive mode, as these clusters tended to display an age-related increase or decline in metabolites, which are likely to be sensitive to aging. Notably, metabolites in the ESI− mode in clusters 8 and 9 exhibited a gradual increasing or decreasing trend with increasing age (Figure 2A), and the same was true for clusters 3 and 4 in the ESI+ mode (Supplementary Figure 2A).

To further narrow down the age-related metabolites, the PLS-DA score between two points in time (25 and 100 weeks) was plotted for both the ESI− and ESI+ modes. This revealed a clear separation between the two points in time (Figure 2B), which was validated by the permutation test, as reflected by that all R2X and Q2 values on the left were lower than the original points on the left (Figure 2C); the same was true for the positive mode (Supplementary Figure 2B, 2C). In total, 1,251 metabolites in ESI− mode and 2,907 metabolites in ESI+ mode were selected by VIP (VIP > 1.0) based on PLS-DA score, of which 1,091 in ESI− mode and 1,559 in ESI+ mode overlapped with the time-series data (Figure 2D and Supplementary Figure 2D).

We next attempted to determine if there were conserved changes in urinary metabolite profiling and conducted a parallel analysis in the test group. Using the “80% rule” filter criteria of primary data in the test group, we derived a list of 9,008 metabolites in the negative mode and 24,951 metabolites in the positive mode for subsequent multivariate statistical analysis. Similar results were obtained for classification trends by the PLS-DA trajectory analysis and PLS-DA score plot of the two time points (Supplementary Figure 3A–3F), suggesting the repeatability of our data.

### Screening of aging-related metabolites based on machine learning

**Figure 3:**
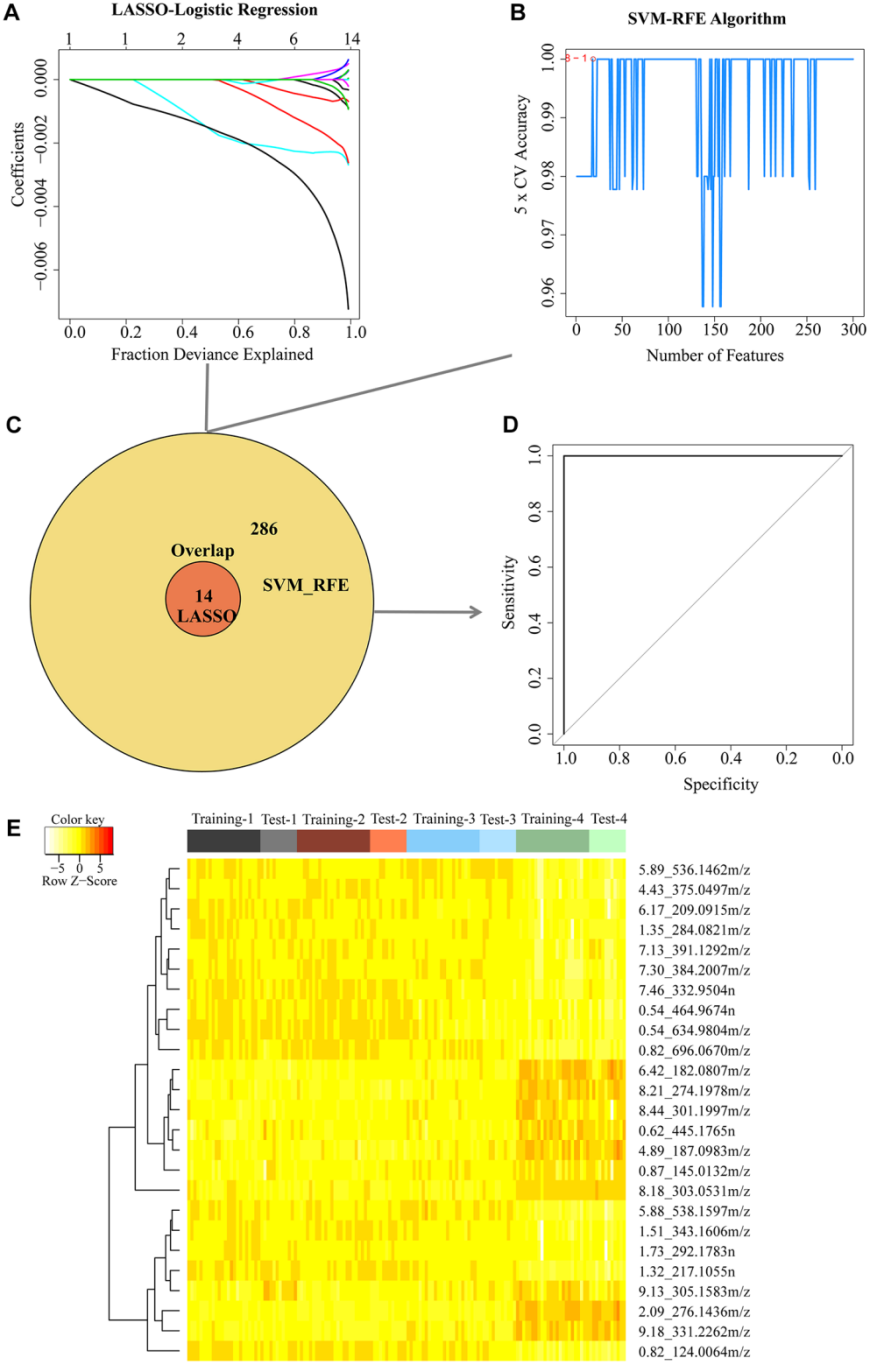
**Two algorithms based on machine learning were used for feature selection in the negative mode.** (**A**) Least Absolute Shrinkage and Selector Operation (LASSO) algorithm in the training group. (**B**) Support Vector Machine-Recursive Feature Elimination (SVM-RFE) algorithm in the training group. (**C**) Venn diagrams demonstrated the overlap of metabolites in combination with two algorithms. (**D**) 14 candidate metabolites detected in the training group were validated in the test group using receiver operator characteristic (ROC) curve analysis. (**E**) Cluster analysis of 25 metabolites in both the negative and positive mode simultaneously selected from the LASSO and SVM-RFE algorithms in the training group.

Considering the data characterized by small sample sizes with high dimensionality, we next combined two different machining learning algorithms to select the aging-related metabolites for classifying 25 and 100 weeks based on 1,091 metabolites in ESI− mode or 1,559 metabolites in ESI+ mode. First, we used the LASSO algorithm to narrow down the range of aging-related metabolites in the training cohort (Figure 3A and Supplementary Figure 4A). For the LASSO penalized regression model, the variables were determined by finding the lambda (λ) with the smallest classification error. As a result, a total of 14 metabolites in ESI− mode and 16 metabolites in ESI+ mode were screened. We also applied the SVM-RFE algorithm to select aging-related metabolites (Figure 3B and Supplementary Figure 4B). The SVM-RFE algorithm selects a feature by eliminating variables one by one based on the feature value ranks computed by the SVM classifier. To obtain more data generated by the intersection of the two methods, we selected the top 300 metabolites based on feature value ranks in the ESI− or ESI+ mode. In combination with the LASSO and SVM-RFE algorithms, 300 metabolites in ESI− mode and 305 metabolites in ESI+ mode related to aging were identified by the union, 14 of which in ESI− mode and 11 in ESI+ mode as candidate metabolites were selected simultaneously by two algorithms (Figure 3C and Supplementary Figure 4C). In addition, we applied these candidate metabolites to predict the aging status in the test cohort using receiver operating characteristic analysis. We found that these metabolites perfectly separated the young and old status in the test cohort, and the area under the curve was 1 in either the ESI− or ES1+ mode (Figure 3D and Supplementary Figure 4D), suggesting that the 25 candidate metabolites could be good aging predictors. The 25 candidate metabolites are shown in the heat map (Figure 3E), reflecting the dramatic metabolic changes at the old stage.

### Identification of aging-associated biomarkers

**Figure 4:**
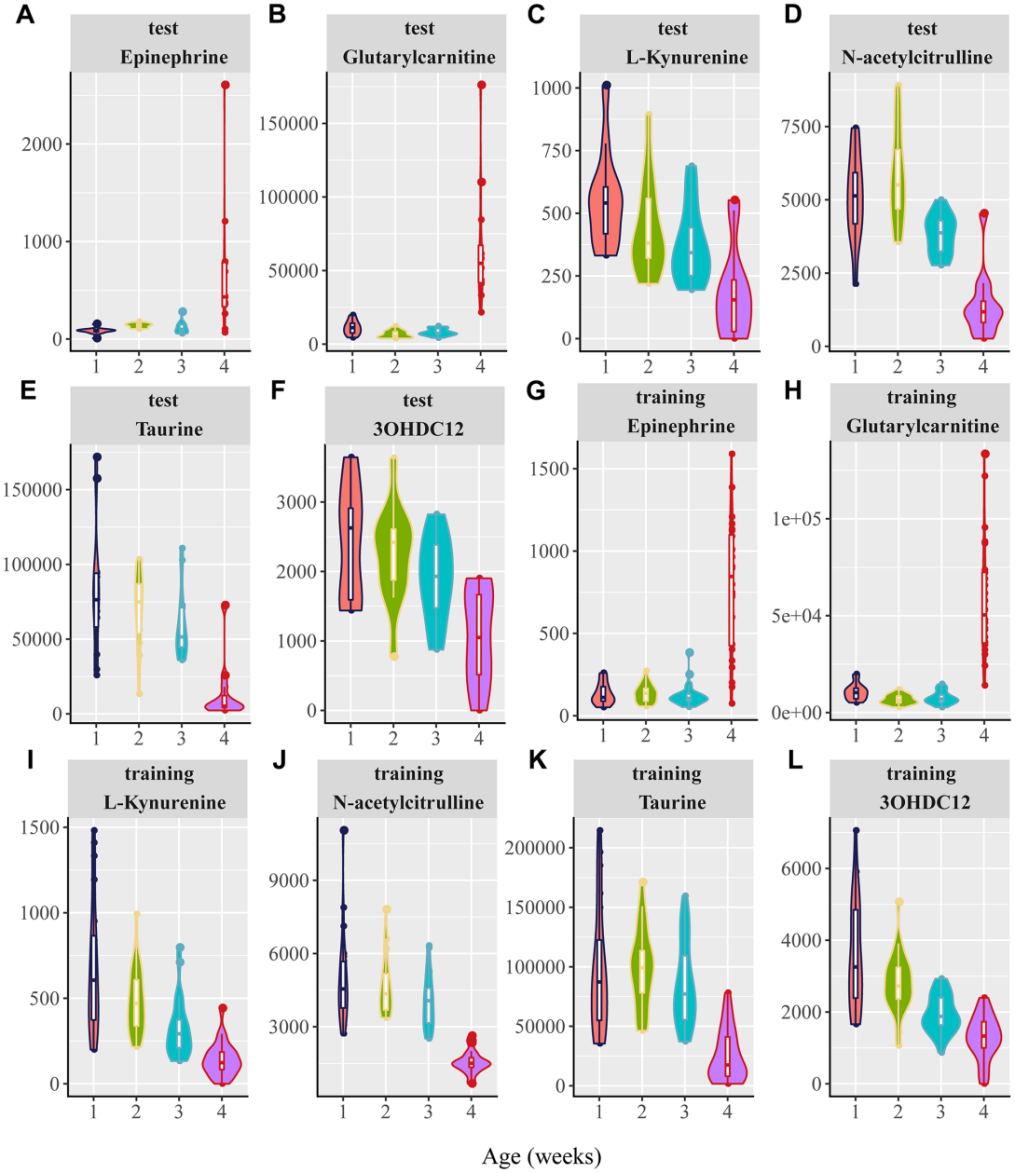
**Violin plots for the six potential biomarkers abundance with aging.** 1, 2, 3, and 4 in the X axis are represented as 20, 25, 50, 75, and 100 weeks, respectively. (**A**–**F**) Normalized abundance of metabolites in both the negative and positive modes in the test group, including (**A**) epinephrine, (**B**) glutarylcarnitine, (**C**) L-kynurenine, (**D**) N-acetylcitrulline, (**E**) taurine, and (**F**) 3-hydroxydodecanedioic acid (3OHDC12). (**G**–**L**) Normalized abundance of metabolites in the negative and positive modes in the training group, including (**G**) epinephrine (**H**) glutarylcarnitine, (**I**) L-kynurenine, (**J**) N-acetylcitrulline, (**K**) taurine, and (**L**) 3-hydroxydodecanedioic acid (3OHDC12). (*P* < 0.0001 for the comparisons between 25 and 100 weeks for the six candidate metabolites in both the test and training groups; one-way ANOVA).

In this study, 25 candidate metabolites related to aging biomarkers were confirmed by comparing their retention times, exact mass, and tandem mass spectrometry fragmentation patterns with those of the standards using MassFragment software. As a result, a total of six metabolites were identified (Table 1). The abundance of these metabolites in both the positive and negative modes is shown in Figure 4A–4L. Epinephrine and glutarylcarnitine levels in rats aged 25 weeks were remarkably higher than those in rats aged 100 weeks, whereas L-kynurenine, N-acetylcitrulline, taurine, and 3-hydroxydodecanedioic acid in rats aged 25 weeks were robustly lower than those in rats aged 100 weeks, in both the training and test cohorts (Figure 4A–4L). Of particular interest, we found that these identified metabolites were in cluster 8 or 9 in the negative mode and in cluster 3 or 4 in the positive mode (Table 1), indicating that these identified metabolites can be reliable aging biomarkers reflecting the aging trajectories.

**Table 1:** Urinary biomarkers identified in the negative and positive modes.

**Mode**	**Ion**	**Compound ID**	**Formula**	**Identified metabolites**	**VIP**	**Cluster**
−	[M-H]−	0.54_464.9674n	Unknown	Unknown	1.422	9
−	[M-H]−	0.54_634.9804m/z	Unknown	Unknown	1.210	9
−	[M-H]−	0.82_124.0064m/z	C2H7NO3S	Taurine	15.06	9
−	[M-H]−	0.82_696.0670m/z	Unknown	Unknown	1.111	9
−	[M-H]−	0.87_145.0132m/z	C5H6O5	Unknown	1.945	8
−	[M-H]−	1.35_284.0821m/z	Unknown	Unknown	1.105	9
−	[M-H]−	4.43_375.0497m/z	Unknown	Unknown	2.097	9
−	[M-H]−	5.89_536.1462m/z	Unknown	Unknown	1.919	7
−	[M-H]−	6.42_182.0807m/z	C9H13NO3	Epinephrine	1.647	8
−	[M-H]−	7.13_391.1292m/z	C22H20N2O5	C22H20N2O5	1.376	8
−	[M-H]−	7.46_332.9504n	Unknown	Unknown	1.508	9
−	[M-H]−	8.18_303.0531m/z	C14H10O5	C14H10O5	1.610	8
−	[M-H]−	8.44_301.1997m/z	C16H30O5	C16H30O5	1.467	8
−	[M-H]−	9.13_305.1583m/z	Unknown	Unknown	4.026	8
+	[M+H]+	0.62_445.1765n	Unknown	Unknown	1.350	4
+	[M+H]+	1.32_217.1055n	C8H15N3O4	N-acetylcitrulline	3.116	3
+	[M+H]+	5.88_538.1597m/z	Unknown	Unknown	2.164	3
+	[M+H]+	1.51_343.1606m/z	Unknown	Unknown	1.523	3
+	[M+H]+	1.73_292.1783n	C12H22O5	3-Hydroxydodecanedioic acid	2.380	3
+	[M+H]+	2.09_276.1436m/z	C12H21NO6	Glutarylcarnitine	10.86	4
+	[M+H]+	6.17_209.0915m/z	C10H12N2O3	L-Kynurenine	1.147	3
+	[M+H]+	7.30_384.2007m/z	Unknown	Unknown	1.155	3
+	[M+H]+	8.21_274.1978m/z	Unknown	Unknown	1.139	4
+	[M+H]+	4.89_187.0983m/z	Unknown	Unknown	1.444	4
+	[M+H]+	9.18_331.2262m/z	C21H30O3	Unknown	5.441	4

### Aging-associated biomarkers and tumor occurrence in rats

**Figure 5:**
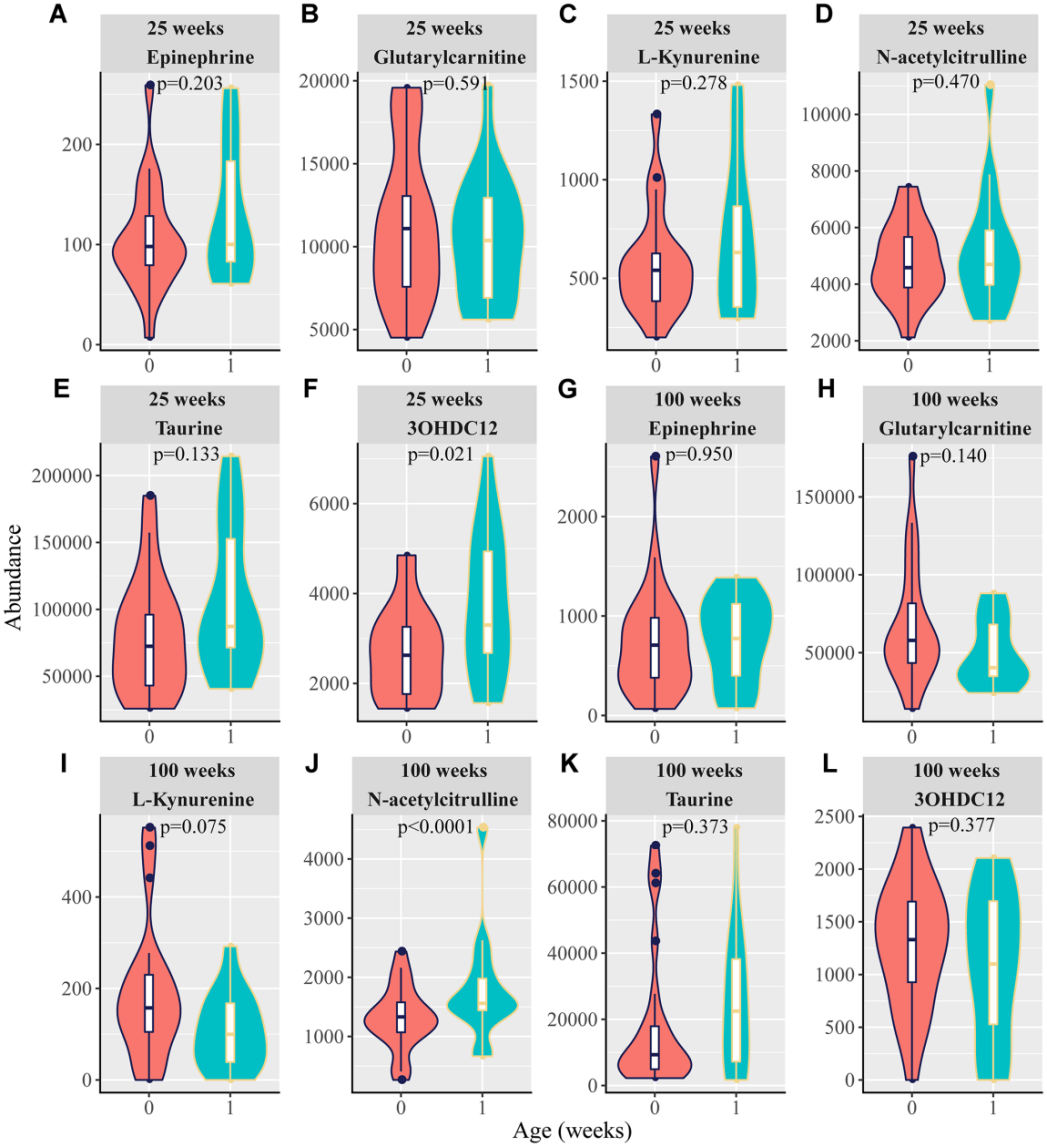
**Violin plots for the six potential biomarkers abundance between tumor-free rats and predicting tumor-bearing rats or tumor-bearing rats.** At 25 weeks, 0 and 1 in the X-axis are represented as tumor-free rats and tumor-bearing rats, respectively. At 100 weeks, 0 and 1 in the X-axis are represented as tumor-free rats and tumor-bearing rats, respectively. (**A**–**F**) Normalized abundance of metabolites in both the negative and positive modes at 25 weeks, including (**A**) epinephrine, (**B**) glutarylcarnitine, (**C**) L-kynurenine, (**D**) N-acetylcitrulline, (**E**) taurine, and (**F**) 3-hydroxydodecanedioic acid (3OHDC12). (**G**–**L**) Normalized abundance of metabolites in both the negative and positive modes at 100 weeks, including (**G**) epinephrine (**H**) glutarylcarnitine, (**I**) L-kynurenine, (**J**) N-acetylcitrulline, (**K**) taurine, and (**L**) 3-hydroxydodecanedioic acid (3OHDC12). (*p* < 0.05 was considered statistically significant; *t*-test).

Given that we observed that many rats carried spontaneous tumors during the long-term feeding period (Table 2), it is interesting to determine whether there are mechanistic links between aging and tumors. Although no tumors were found in 25-week-old rats, some developed tumors after 25 weeks. Therefore, we first selected rats aged 25 weeks to explore the relationship between the identified aging-related metabolites and the occurrence of tumors in the future (predicting tumor). Epinephrine, glutarylcarnitine, L-kynurenine, taurine, and N-acetylcitrulline did not differ between tumor-free rats and tumor-bearing rats, whereas the 3-hydroxydodecanedioic acid level was higher in the tumor-bearing rats than in the tumor-free rats (Figure 5A–5F). To further investigate the potential contribution of aging-related metabolites to tumors, the rats aged 100 weeks were analyzed. We found that, compared to that in tumor-free rats, epinephrine, glutarylcarnitine, L-kynurenine, taurine, and 3-hydroxydodecanedioic acid levels were not different, but N-acetylcitrulline in the tumor-bearing rats was obviously increased (Figure 5G–5L).

**Table 2:** Pathological tumor classification determined by a veterinary pathologist in rats.

**Location**	**Tumor**	**25 weeks (*n* = 36)**	**100 weeks (*n* = 36)**
		**Predicting tumor (*n* = 16)**	**Tumor (*n* = 14)**
Head	Hepatocellular carcinoma	1	0
	Pituitary adenoma	2	3
	Unspecified tumor	1	2
Thoracic cavities	Lymphoma	2	0
	Squamous cell carcinoma	0	1
	Myxoma (unspecified tumor)	1	0
Abdominal cavities	Hepatocellular carcinoma	1	2
	Adenocarcinoma	1	1
	Lymphoma	1	0
	Mucoid carcinoma	0	1
	Epitheliogenic tumor (unspecified tumor)	0	1
Subcutaneous	Squamous cell carcinoma	1	2
	Subcutaneous fibrosarcoma	1	0
	Fibroma	1	0
	Myxoma (unspecified tumor)	3	2
	Total numbers of tumors	16	14

## DISCUSSION

Multiple studies have sought to identify genetic and metabolite factors that affect lifespan [2–4, 6, 13–15]. Extending this prior work, in this study, we used high-performance LC/MS to dynamically detect urine metabolites with increasing age in the training cohort and selected 25 candidate metabolites related to aging by combining four algorithms, including VIP, time-series, LASSO, and SVM-RFE. We further validated the candidate metabolites using the test cohort, in which these metabolites could stratify the test data into two distinct subgroups with young or old age. Finally, we identified six metabolites from 25 candidates, some of which were not previously associated with aging, which offers a novel view of the potential pathways that counteract aging. In addition, we assessed the associations between the identified aging-related metabolites and tumor occurrence.

To gain insight into the mechanism underlying metabolism and aging and to identify targets for delaying aging, identifying reliable biomarkers related to aging may be an effective strategy. The emerging algorithms are likely to contribute to biomarker discovery, especially machine learning, including LASSO and SVM. Indeed, many studies have applied machine learning models to disease diagnosis and prognosis, as well as marker identification. Jiliang et al., found that MSEH, a three-CpG-based signature, favors recurrence prediction in patients with early stage hepatocellular carcinoma based on the LASSO-logistic regression and SVM-RFE algorithms [16]. Liang et al., (2016) used machine learning to identify 15 metabolites in urine metabolomics as hepatocarcinoma markers [17]. Furthermore, previous studies have applied machine learning to reveal metabolites associated with age in a healthy adult population using cross-sectional data [18, 19]. However, the application of machine learning to dynamically screen urine metabolites related to aging in a cohort is still lacking. In this study, we established two cohorts of rats, starting from early adulthood to old age. We selected and identified six candidate metabolites in combination with multiple algorithms, including VIP, time-series analysis based on soft cluster, LASSO, and SVM-RFE. It is interesting to note that although we set loose restrictions in the time-series analysis, the six identified aging-related metabolites were found in clusters 8 and 9 in the ESI− mode or in clusters 3 and 4 in the ESI+ mode, which displayed semi-U-shaped or semi-parabolic-shaped aging trajectories characterized by a gradual increasing or decreasing trend with increasing age. We further found that the abundance of the six metabolites was remarkably higher or lower at the age of 100 weeks than at 25 weeks, although we did not set a *p-value* limit in our screening process, suggesting that machine learning may be an alternative statistical tool to the traditional *p* algorithm.

Consistent with prior reports relating to taurine levels declining with age in the cornea [20], we also found a decrease with age in urine. Taurine, a ubiquitous sulfur-containing amino acid, regulates mitochondrial function, inhibits mitochondrial ROS production, and suppresses inflammation [21–24], indicating its potential antiaging function. Accordingly, taurine transporter knockout mice display abnormal skeletal muscle and accelerated skeletal muscle aging [25]. In addition, taurine supplementation has been reported to delay aging-associated diseases, including chronic heart failure [26], diabetes [27], and endothelial dysfunction [28].

Additional metabolites related to aging included L-kynurenine, which is a major degradation metabolite of tryptophan. Inhibition of tryptophan conversion to l-kynurenine is sufficient to prolong lifespan [29, 30]. A previous study showed that the plasma tryptophan/L-kynurenine ratio increased with age. In contrast, we found that L-kynurenine abundance decreased with age in urine. We speculate that the decrease in L-kynurenine may be due to the decline of tryptophan with age, suggesting that the ratio of tryptophan/L-kynurenine may be more effective as an aging-related marker than L-kynurenine alone.

N-acetylcitrulline is a citrulline metabolite involved in the arginine synthesis pathway [31]. Although a causal link between lifespan and acetylcitrulline has not been established, the arginine synthesis pathway involved is shown to be implicated in longevity. Arginine supplementation increased the lifespan of *C. elegans* by activating the DAF-16/FOXO or SKN-1/Nrf2 stress response pathways [32]. Moreover, intracellular L-arginine supplementation enhances the survival of T cells and favors antitumor responses [33].

Epinephrine is the main hormone in the adrenal medulla [34]. Although data relating epinephrine to longevity are scant, a substantial body of research has implicated epinephrine function in increasing memory [35–37], regulating blood pressure [38, 39], and lowering blood sugar [40]. The link between other metabolites, including glutarylcarnitine and 3-hydroxydodecanedioic acid, and aging has not been established previously. Glutarylcarnitine is formed through the combination of glutaryl-CoA and carnitine and participates in protein biosynthesis [41]. 3-Hydroxydodecanedioic acid is an organic compound known as a medium-chain fatty acid. Current research on glutarylcarnitine and 3-hydroxydodecanedioic acid has focused on its relationship with glutaric aciduria type I and the disorder in fatty acid oxidation, respectively [42–47]. Further research is needed to investigate the relationship between glutarylcarnitine and 3-hydroxydodecanedioic acid metabolism and lifespan.

Because tumors are closely related to aging, delaying tumor occurrence over the life course would appear to strongly favor longevity [48–51]. Therefore, we also assessed the association between the identified aging-related metabolites and tumor occurrence. Among the six candidate metabolites, compared with tumor-free rats, we found that 3-hydroxydodecanedioic acid and N-acetylcitrulline levels were increased in predicting tumor-bearing rats at 25 weeks and in tumor-bearing rats at 100 weeks, respectively. Consistent with our findings, N-acetylcitrulline has been shown to be associated with the incidence of mammary cancers [52] and prostate cancer [53, 54]. Notably, we observed that 3-hydroxydodecanedioic acid was related to predicting tumor occurrence at 25 weeks, but not tumor occurrence at 100 weeks, implying that 3-hydroxydodecanedioic acid may not fit well in tumor diagnosis. In addition, it is interesting to note that in contrast to an increase in tumor rats, 3-hydroxydodecanedioic acid and N-acetylcitrulline decreased with increasing age, indicating a complexity related to the aging of the tumor. Because of the different and specific metabolic microenvironment and biological metabolic processes, it is reasonable to speculate that most aging-related markers are independent of spontaneous tumors, although some aging-related markers can be influenced by tumors in this study.

Our study had several limitations. Only male sex was investigated in this experiment, although sex is known to be involved in different responses to aging and metabolic homeostasis [55]. In addition, we identified six aging-related metabolites that have not yet been validated in human samples; thus, our observations should be considered as hypothesis-generating that only provides a potential application of these biomarkers in humans. Further investigations of these candidate metabolites in human cohorts of different ages and sexes are warranted.

In summary, we screened six metabolites as known or novel aging-related markers in two long-term rat experiments in combination with different algorithms and applied machine learning. These findings may pave the way for the prediction of aging and targeting of anti-aging agents. Further research is needed to confirm the mechanism underlying the identified metabolites and aging, as well as its investigation in the human population.

## MATERIALS AND METHODS

### Animals and diets

A total of 90 male Wistar rats aged 8 weeks were supplied by the Vital River Laboratory Animal Technology Company LTD (Beijing, China). After three days of adaptation, all rats were randomly assigned to three groups with different purified diets based on the standard purified rodent diet (AIN-93G) paradigm: regular control diet (AIN-93G diet), high-protein diet, and high-fat diet. A high-protein feed was developed by adjusting the content of casein, corn starch, and sucrose according to AIN-93G. The high-fat feed was developed by adjusting lard, corn starch, sucrose content, and micronutrient content according to AIN-93G. In this study, we combined the first two diet groups as the training group and the other diet group as the test group. We aimed to investigate aging trajectories and aging-related metabolites, regardless of diet influence. All animals were housed in a single stainless-steel cage. The rats were fed daily, and food intake was recorded once every one–two days. To ensure palatability, pellet chow was produced every 3 months and stored at -20°C. Body weight was monitored weekly in the morning. Rats were maintained in a light/dark cycle for 12/12 h at a temperature of 21 ± 2°C and humidity of 50%–60% under pathogen-free conditions. In this long-term experiment, rats were euthanized when they were considered inharmonious with continued survival based on the euthanasia criteria from the AAALAC guidelines (data not shown). All protocols were approved by the Medical Ethics Committee of Harbin Medical University (Harbin, China) and were performed in accordance with the Guide for Care and Use of Laboratory Animals (ethics number: 2015138).

### Collection of urine

Animal urine was dynamically collected at four time points using metabolic cages (training group: *n* = 24 each time, test group: *n* = 12 each time; age = 25, 50, 75, and 100 weeks). A 24-h urine was collected and then divided into several tubes and stored at −80°C for further measurement after being centrifuged at 3000 rpm for 15 min at 4°C.

### Metabolomics analysis of rat urine by UPLC/Q-TOF MS

Urinary untargeted metabolomics analysis was performed using ultra-performance liquid chromatography and quadrupole time-of-flight tandem mass spectrometry (UPLC-Q-TOF-MS) is described as follows:

### Chemicals and reagents

The following reagents used are high-performance liquid chromatography (HPLC) grade: acetonitrile (Fisher Scientific Los Angeles, CA, USA), methanol (Fisher Scientific Los Angeles, CA, USA), formic acid (Sigma-Aldrich, St Louis, MO, USA) and leucine-enkephalin (Sigma-Aldrich, St Louis, MO, USA). Ultrapure water was prepared using an Ultra Clear System (Siemens Water Technologies, Nuremberg, Germany).

### Sample preparation

Urine samples were thawed at 4°C, vortex-mixed for 1 min, diluted 1:11 (v/v) with water, mixed by vortexing for 1 min, and centrifuged at 12 000 × *g* for 10 min at 4°C. The supernatant was transferred to an autosampler vial. All samples were taken equally and mixed well to prepare quality control (QC) samples.

### Chromatography

Chromatographic separation was carried out using a Waters ACQUITY UPLC System (Waters, Milford, MA) with an HSS T3 column (100 × 2.1 mm; id. 1.7 um; Waters). A 2 μL urine sample was injected into the column maintained at 35°C at a flow rate of 0.45 mL/min through a 4°C autosampler. The mobile phase included solutions A (0.1% formic acid in water) and solution B (pure acetonitrile). The elution gradients were as follows: 2% B for 0–0.5 min, 2%–20% B for 0.5–7 min, 20%–35 % B for 7–8 min, 35%–70% B for 8–9.5 min, 70%–98% B for 9.5–11 min, 98% B for 11–13 min, 2% B for 13–14 min, 2% B for 14–16 min in the positive and negative ion modes. Acetonitrile as a blank and QC were injected every 15 samples, respectively, and urine samples at different time points were run alternately to eliminate the bath effect.

### Mass spectrometry

Mass spectrometry was performed using a Waters Micromass Q-TOF (Waters, Manchester, UK) equipped with electrospray ionization (ESI) in the positive and negative modes. The parameters were set as follows: capillary voltage, 0.5 kV; sample cone voltage, 60 V; collision energy, 6 eV; source temperature, 110°C; desolvation gas (nitrogen) flow, 900 L/h; desolvation temperature, 450°C; cone gas (nitrogen) flow, 50 L/h; collision gas, argon; and MCP detector voltage, 2550 V. The scan mass range was 50–1200 m/z for 0–16 min. A concentration of 200 pg/μL leucine-enkephalin was used as the mass spectrum correction ion lock mass ([M+H] = 556.277 1, [M–H] = 554.261 5). Scan frequency was set at 0.2 s every 15 s scans for correction. The MS/MS spectra of the potential biomarkers were obtained by UPLC-QTOF-MS/MS.

### Data processing

Raw data were uploaded to the QI (version 2.1; Waters Corporation, Milford, MA, USA) for peak detection and alignment, and were further analyzed using EZ info software (version 2.0; Umetrics AB, Umea, Sweden) and SIMCA-P (version 12.0; Umetrics AB, Umea, Sweden). To eliminate the variations in urine concentrations of different rats, normalization of each ion intensity to the total ion intensity for each chromatogram was performed. Prior to multivariate statistical analysis, the “80% rule” filter criteria as a common method were used to filter missing values that arise from technology or sample and impact the statistical analysis in metabonomics research [56]. Multivariate statistical analysis was performed based on the Pareto-scaled or UV-scaled data. Principal component analysis (PCA) was conducted by combining QC samples and all experimental samples to evaluate the reproducibility and quality of the data. Partial least squares-discriminant analysis (PLS-DA) was applied to visualize the maximal separation at different time points. 200 Random permutation testing was carried out to avoid over-fitting of PLS-DA models using SIMCA-P software (version 12.0; Umetrics AB, Umea, Sweden). Metabolites with variable importance in the project (VIP) value above 1.0, were selected for analysis.

The metabolites were first identified using Progenesis QI software linked to online databases, such as ChemSpider (http://www.chemspider.com), the Human Metabolome Database (HMDB) (http://www.hmdb.ca), METLIN (http://metlin.scripps.edu/) and Pubchem (http://pubchem.ncbi.nlm.nih.gov/) (the tolerance mass was set at 10 ppm). The MassFragment™ application manager (MassLynx v4.1, Waters Corp., USA) was used to facilitate the MS/MS fragment ion analysis process based on chemically intelligent peak-matching algorithms. Finally, the biomarkers were further confirmed by standard substances based on both retention times and MS/MS spectra.

### Pathology examinations

Tumors were fixed in 4% paraformaldehyde and embedded in paraffin. Paraffin sections (6 μm thick) were stained with hematoxylin and eosin (H&E). Histological examinations were performed by pathologists who were blinded to the study groups.

### Statistical analysis

Unless otherwise stated, data are presented as the mean ± SD. Food intake and body weight trajectories were plotted using generalized additive models to fit a smooth curve, using R version 3.6.2. The packages of Mfuzz, glmnet, and e1071 were loaded to calculate time-series, LASSO, and SVM-RFE, respectively, using R software. Receiver operator characteristic (ROC) curve analysis was performed by R. Differences were analyzed by one-way ANOVA test or nonparametric Kruskal–Wallis test and *p* value < 0.05, which was considered statistically significant.

## Supplementary Materials

Supplementary Figures
