# Supplementary material for: Aging-related markers in rat urine revealed by dynamic metabolic profiling using machine learning
Source: Aging (Albany NY). 2021 May 19;13(10):14322–41. doi: 10.18632/aging.203046 (PMC8202887; doi:10.18632/aging.203046)
Supplement: Supplementary Figures [file aging-13-203046-s001.pdf]

## SUPPLEMENTARY FIGURES

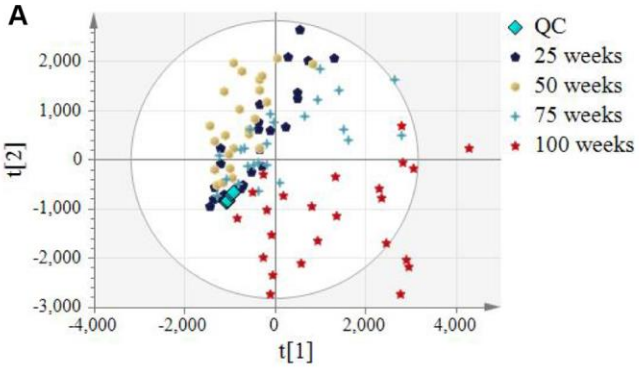


**Supplementary Figure 1. PCA scores plots of urine obtained from the UPLC-Q-TOF-MS.** PCA score plots including quality control (QC) samples and experimental samples of time data in (**A**) the positive mode and (**B**) the negative mode.

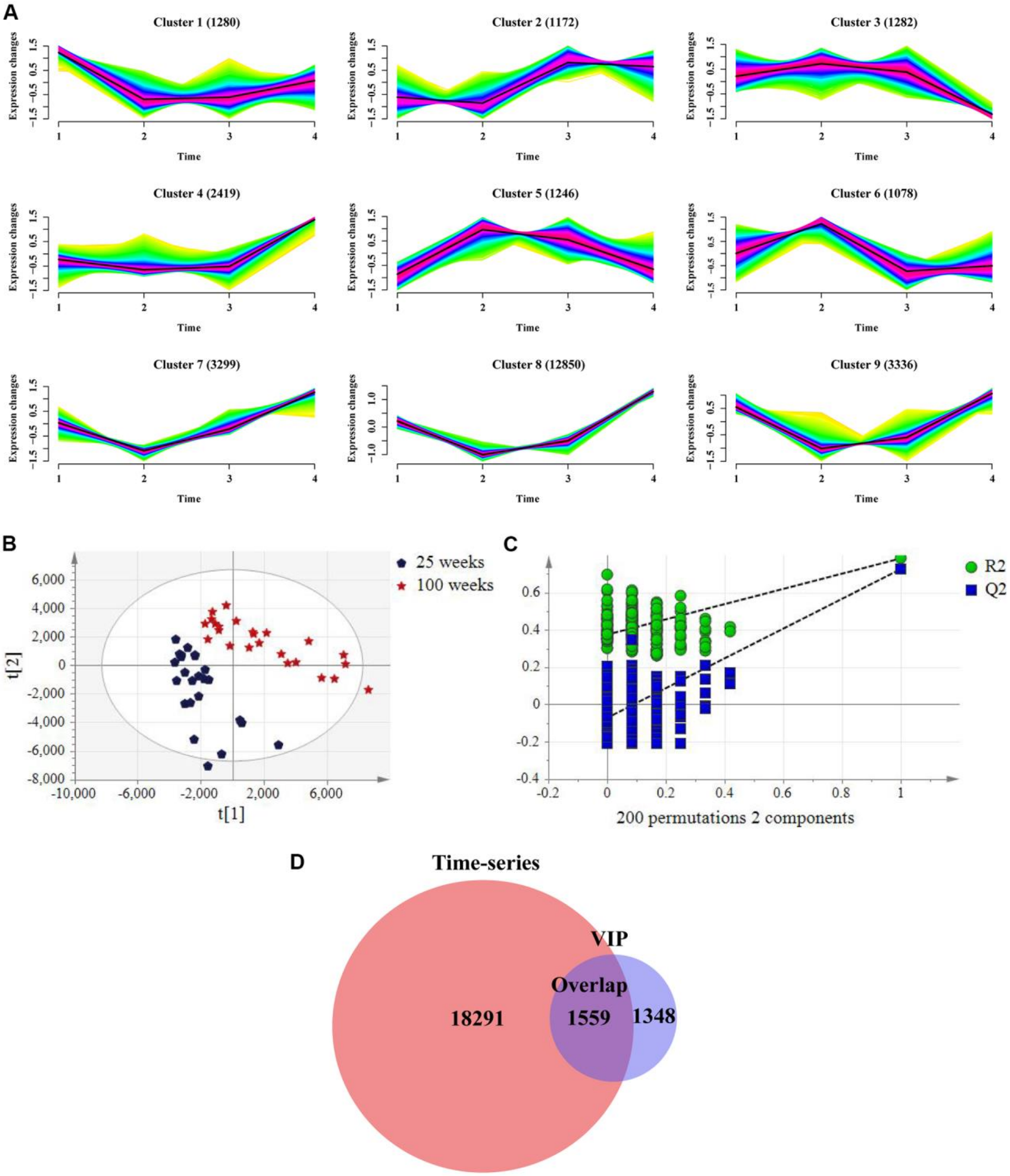


**Supplementary Figure 2. Screening metabolites in combination with time-series analysis and variable importance in the project (VIP) value in the positive mode in the training group.** (**A**) Metabolic aging trajectories. Fuzzy c-mean clustering of all 27962 metabolite abundances. Average trend of clusters is shown as a black line. (**B**) PLS-DA scores plots of rat urine at 25 weeks and 100 weeks. (ESI+, R2Y= 0.784, Q2 = 0.727). (*n* = 24 each time point). (**C**) Permutation test with 2 components of PLS-DA score plot. (**D**) Venn diagrams demonstrated the overlap of metabolites in combination with two algorithms.

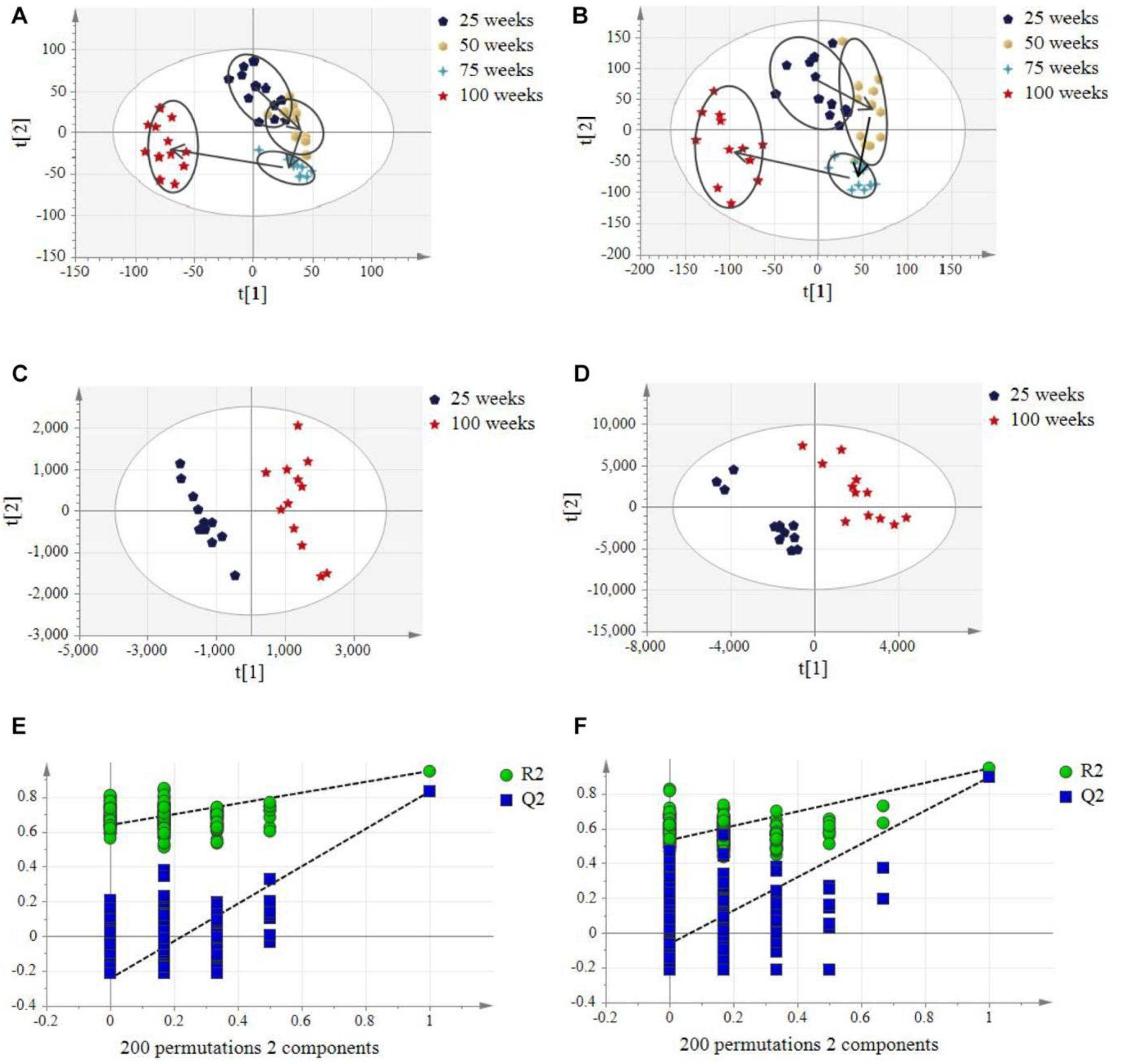


**Supplementary Figure 3. Aging trajectories in the negative and positive modes in the test group.** (**A**–**B**) PLS-DA scores plots of urine obtained from the UPLC-Q-TOF-MS with aging in (A) left: the negative mode, and (B) right: the positive mode in the test group. (*n* = 12 each time point) (**C**–**D**) PLS-DA scores plots at 25 weeks and 100 weeks in (C) left: the negative mode, and (D) right: the positive mode in test group. (ESI−, R2Y= 0.95, Q2 = 0.832; ESI+, R2Y= 0.946, Q2 = 0.895). (*n* = 24 each time point). (**E**–**F**) Permutation test with 2 components of PLS-DA scores plots related to Figure C and D in (E) left: the negative mode, and (F) right: the positive mode in the test group.

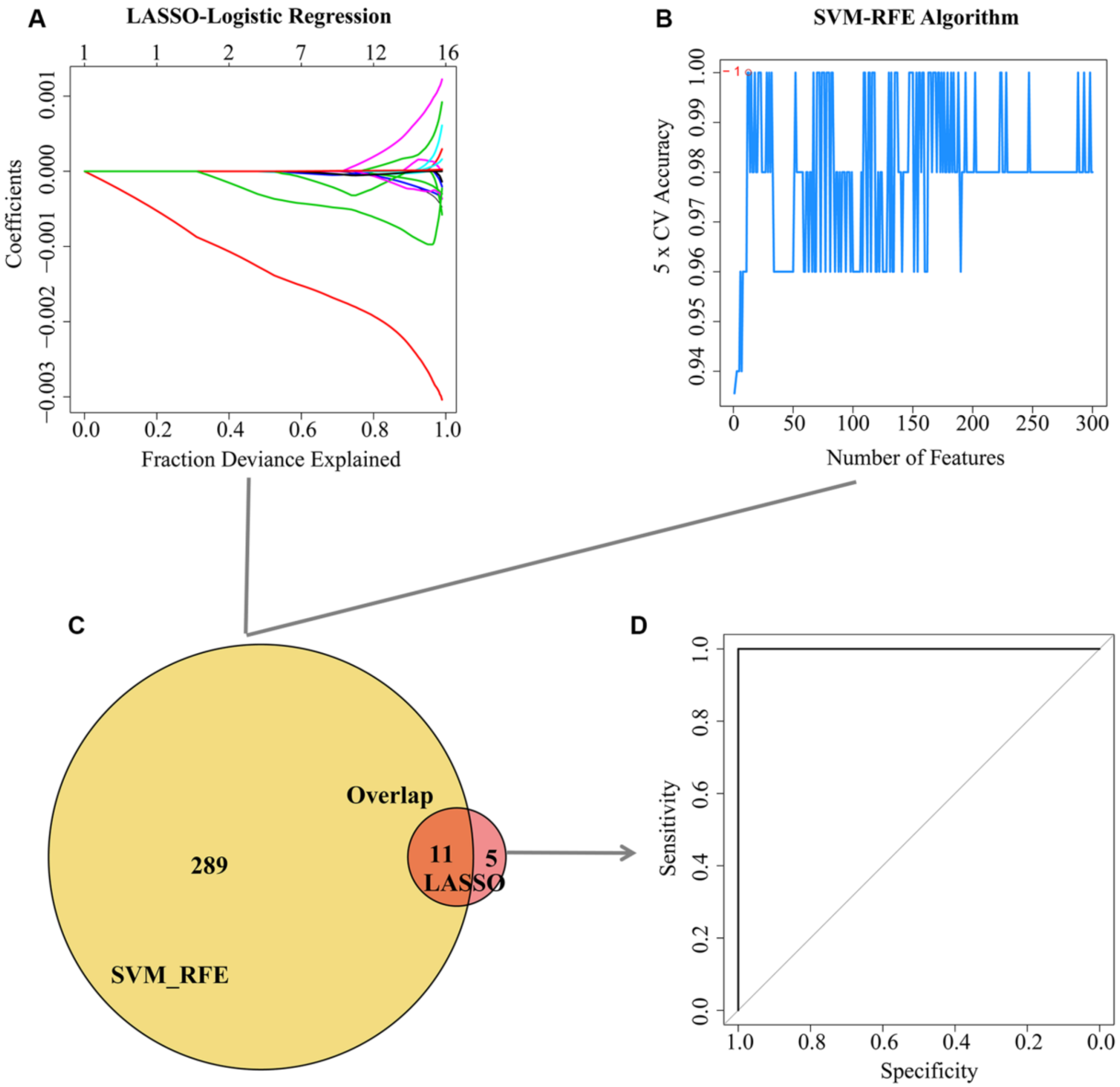


**Supplementary Figure 4. Two algorithms based on machine learning were used for feature selection in the positive mode.** (**A**) Least Absolute Shrinkage and Selector Operation (LASSO) algorithm in the training group. (**B**) Support Vector Machine-Recursive Feature Elimination (SVM-RFE) algorithm in the training group. (**C**) Venn diagrams demonstrated the overlap of metabolites in combination with two algorithms. (**D**) 11 candidate metabolites detected in the training group were validated in the test group using receiver operator characteristic (ROC) curve analysis.
